# Supplementary material for: Type I interferon signature strength correlates with alloimmunization-associated transcriptomic programs in systemic lupus erythematosus: A multi-cohort analysis
Source: PLoS One. 2026 Sep 15;21(9):e0347443. doi: 10.1371/journal.pone.0347443 (PMC13577513; doi:10.1371/journal.pone.0347443)
Supplement: Supplementary file 1 [file pone.0347443.s001.docx]

S1 Table. Selected differentially expressed genes in IFN-high versus IFN-low SLE patients, organized by biological pathway

| Gene | Protein function | log2FC | *p*-Value | FDR q-Valuea |
| --- | --- | --- | --- | --- |
| *IFN-stimulated genes* | | | | |
| *MX1* | Antiviral GTPase; IFN-α/β effector | +2.80 | 2.56×10-17 | 4.13×10-15 |
| *OAS1* | 2’-5’ oligoadenylate synthetase; RNA degradation | +2.68 | 7.84×10-19 | 2.17×10-16 |
| *ISG15* | Ubiquitin-like modifier; antiviral conjugation | +3.38 | 1.93×10-19 | 7.47×10-17 |
| *IFIT1* | Tetratricopeptide repeat; blocks viral RNA | +3.25 | 4.33×10-16 | 5.66×10-14 |
| *RSAD2* | Radical SAM enzyme; restricts RNA virus replication | +3.98 | 1.62×10-15 | 1.80×10-13 |
| *SIGLEC1* | Sialic acid receptor on monocytes; IFN-I marker | +3.56 | 2.24×10-16 | 3.17×10-14 |
| *Alloimmunization-associated genes* | | | | |
| *IFIH1* | MDA5 cytosolic RNA sensor; innate immune activation | +1.78 | 4.44×10-21 | 5.86×10-18 |
| *IRF7* | Master transcription factor for IFN-α/β production | +1.86 | 7.14×10-23 | 8.49×10-19 |
| *DDX58* | RIG-I RNA helicase; innate antiviral sensing | +1.55 | 2.38×10-19 | 8.10×10-17 |
| *Complement activation genes* | | | | |
| *C2* | Complement component *C2*; classical/lectin pathway | +1.01 | 7.56×10-8 | 2.69×10-6 |
| *C1QC* | C1q chain C; classical complement initiation | +0.99 | 4.02×10-5 | 5.58×10-4 |
| *C1QB* | C1q chain B; opsonization and immune complex clearance | +0.76 | 1.88×10-3 | 1.09×10-2 |
| *Plasmablast differentiation genes* | | | | |
| *MZB1* | ER chaperone; antibody secretion in plasmablasts | +1.18 | 1.10×10-4 | 1.24×10-3 |
| *CD38* | Transmembrane glycoprotein; plasmablast/plasma cell marker | +1.09 | 1.07×10-8 | 4.54×10-7 |
| *TXNDC5* | Thioredoxin domain protein; ER stress in B cell differentiation | +0.87 | 2.49×10-4 | 2.34×10-3 |
| *Control pathway gene (downregulated)* | | | | |
| *KLRB1* | CD161; NK cell and T cell surface marker | −0.86 | 8.76×10-7 | 2.27×10-5 |

a Differential expression was assessed using the Welch two-sample t-test on z-scored expression values (IFN-high, n = 49; IFN-low, n = 50). log₂FC = log₂ fold change (mean z-score difference). A total of 665 genes reached significance (FDR < 0.05, |log₂FC| > 0.5): 538 upregulated, 127 downregulated. FDR q-values calculated by the Benjamini–Hochberg procedure. Genes shown are representative of each pathway category.

Abbreviations: ER, endoplasmic reticulum; FDR, false discovery rate; IFN, interferon; log₂FC, log₂ fold change; NK, natural killer; SAM, S-adenosylmethionine; SLE, systemic lupus erythematosus.
